# Residual disease activity in cystic fibrosis lung disease: implications for clinical management and research

**DOI:** 10.1093/ajrccm/aamag370

**Published:** 2026-07-11

**Authors:** Andrea Gramegna, Gianmarco Putti, Gianfranco Alicandro, Marcus A Mall, Francesco Blasi

**Affiliations:** Department of Pathophysiology and Transplantation, Università degli Studi di Milano, Milan, Italy; Respiratory and Cystic Fibrosis Unit, Fondazione IRCCS Ca’ Granda Ospedale Maggiore Policlinico, Milan, Italy; Department of Pathophysiology and Transplantation, Università degli Studi di Milano, Milan, Italy; Department of Pathophysiology and Transplantation, Università degli Studi di Milano, Milan, Italy; Mother and Child Department, Cystic Fibrosis Centre, Fondazione IRCCS Ca’ Granda Ospedale Maggiore Policlinico, Milan, Italy; Department of Pediatric Respiratory Medicine, Immunology and Critical Care Medicine, Charité–Universitätsmedizin Berlin, Berlin, Germany; German Center for Child and Adolescent Health (DZKJ), Partner Site Berlin, Berlin, Germany; German Center for Lung Research (DZL), Associated Partner Site Berlin, Berlin, Germany; Cluster of Excellence ImmunoPreCept, Charité–Universitätsmedizin Berlin, Berlin, Germany; Department of Pathophysiology and Transplantation, Università degli Studi di Milano, Milan, Italy; Respiratory and Cystic Fibrosis Unit, Fondazione IRCCS Ca’ Granda Ospedale Maggiore Policlinico, Milan, Italy

**Keywords:** cystic fibrosis, residual disease activity, CFTR modulators

## Abstract

The advent of cystic fibrosis transmembrane conductance regulator (CFTR) modulators is fundamentally reshaping research and care for people with cystic fibrosis (CF). Despite unprecedented improvements in lung function and predicted survival, a substantial proportion of people with CF continue to exhibit residual disease activity (RDA) across multiple biological and clinical domains. Persistent abnormalities in CFTR function, mucus properties, mucociliary clearance, airway infection, inflammation, and symptom burden indicate that CFTR modulation does not fully normalize airway physiology or eliminate the pathological processes underlying CF lung disease. This review proposes RDA as a conceptual framework for identifying ongoing, potentially modifiable disease processes in people receiving CFTR modulators. Candidate measures across complementary mechanistic and clinical domains are examined, and their potential multidimensional interpretation as a means of supporting more individualized monitoring and treatment decisions is discussed. We consider the implications of RDA for clinical research by identifying key evidence gaps, outlining a stepwise pathway for its definition and validation. We further explore the potential to inform sensitive endpoint selection, enrich study populations, and guide the development of therapies targeting disease processes that persist despite CFTR modulation. As the CF population ages and disease trajectories evolve, care models and research priorities must move beyond conventional measures of disease severity alone. A better understanding and systematic evaluation of RDA may help align long-term clinical management and future trial design with the changing needs of people with CF, while ensuring that therapeutic advances translate into sustained health gains.

**CME will be available for this article at**  https://atsedplus.thoracic.org/course-catalog-list

## Introduction

The introduction of triple-combination cystic fibrosis transmembrane conductance regulator (CFTR) modulators (CFTRm) has fundamentally impacted on the clinical trajectory of people with cystic fibrosis (pwCF), leading to marked attenuation of disease progression and a further increase in life expectancy over the coming years.^1^

Evidence from clinical trials, real-world observational studies, and registries shows significant short- to medium-term improvements in lung function, nutritional status, and patient-reported outcomes.^2-9^ The clinical benefit has also been confirmed in pwCF with advanced lung disease, allowing for deferral of lung transplant in a considerable number of patients.^10^ To date, the integration of real-world evidence from the French compassionate use program,^11^ along with in vitro data and clinical trial results on rare variants,^11^^,^^12^ has made elexacaftor/tezacaftor/ivacaftor (ETI) available to up to 95% of the European cystic fibrosis (CF) population. As a result, life expectancy is projected to move toward that of the general population.^13^ This paradigm shift underscores the growing demand to characterize new clinical trajectories and to reshape prevention strategies and models of care to meet the evolving clinical needs of an aging CF population.

Multiple factors are expected to influence long-term disease trajectories in this era (Figure 1). As life expectancy increases, age-related comorbidities are likely to emerge and gain clinical relevance, contributing to residual morbidity and influencing care management needs.^14^ In parallel, both age and disease severity at the time of initiation of CFTRm represent key determinants of therapeutic response. Adults with established bronchiectasis have limited potential for structural and biological reversal compared with infants and young children who begin therapy before irreversible lung damage has occurred.^15-18^

**Figure 1:**
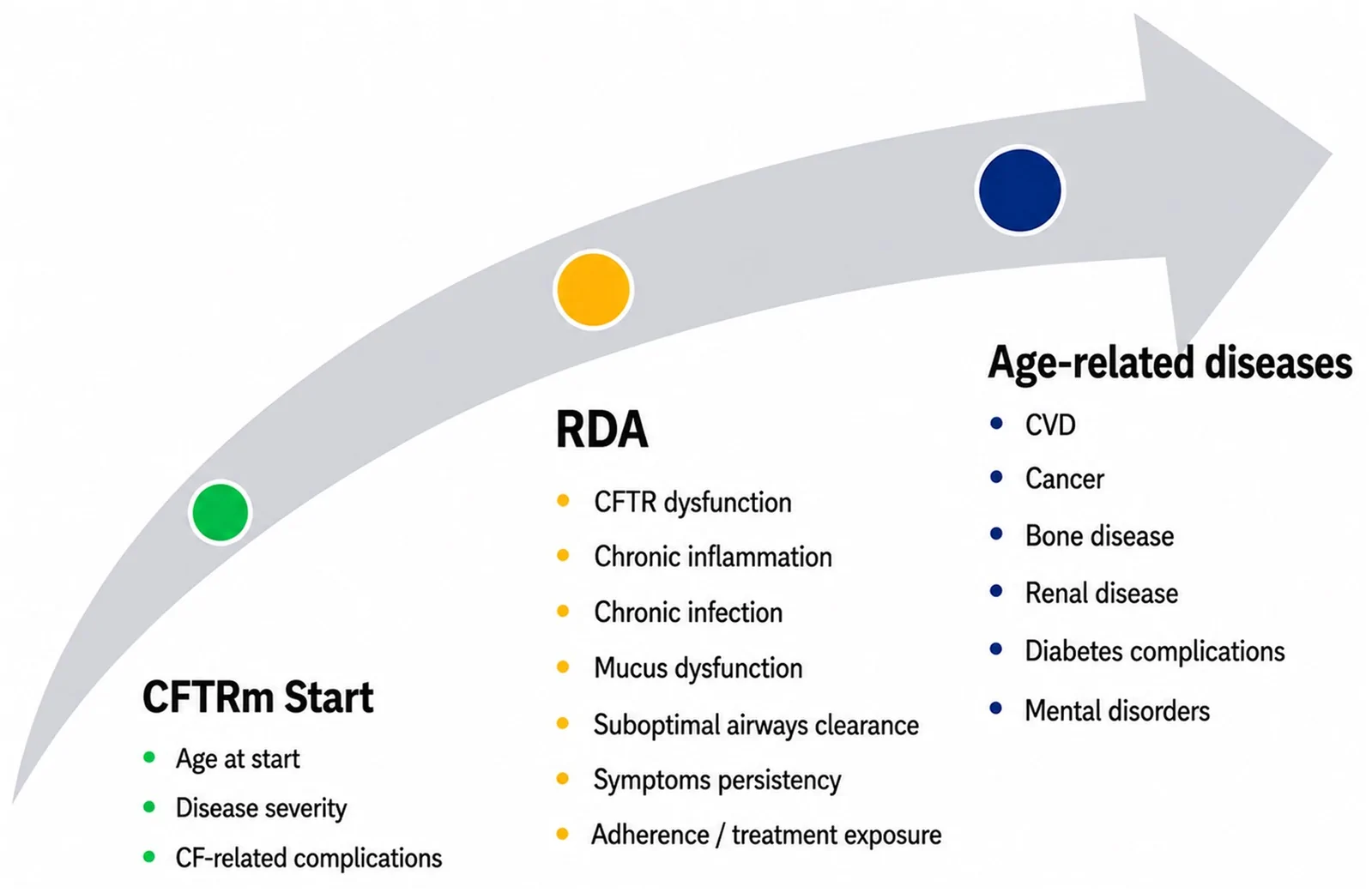
Factors contributing to long-term disease trajectories in the era of CFTR modulators. Abbreviations: CF, cystic fibrosis; CFTR, cystic fibrosis transmembrane conductance regulator; CFTRm, cystic fibrosis transmembrane conductance regulator modulators; CVD, cardiovascular disease; RDA, residual disease activity.

Despite this transformative impact, a substantial proportion of pwCF may continue to exhibit persistence of pathophysiological abnormalities that can contribute to ongoing disease progression despite partial restoration of CFTR function. We propose the term “residual disease activity” (RDA) to describe this continuing and potentially modifiable pathological activity.

This review aims to explore the available evidence on persistent pathophysiological abnormalities following CFTRm and to organize these findings within a multidomain framework of RDA. A secondary aim is to examine the potential clinical relevance of this framework for patient stratification, longitudinal monitoring, and treatment optimization. This review also seeks to identify key evidence gaps, including the absence of a standardized definition of RDA, the lack of a validated assessment tool, and the limited ­evidence supporting RDA-guided clinical decision-making. Addressing these gaps is crucial to move beyond a purely modulator-centered view of disease control and to identify residual, potentially treatable targets that may guide individualized follow-up strategies and therapeutic interventions.

## Definition of disease activity in CF

### Disease activity versus disease severity

Disease activity may be defined as the current intensity of ongoing pathophysiological processes that drive disease progression and that can be potentially suppressed or partially modified by treatment.^19-21^ In other fields as rheumatology, disease activity has traditionally been conceptualized as the potentially reversible inflammatory disease manifestations, in contrast to disease severity, which reflects persistent or irreversible anatomical, physiological, or functional damage accumulated over time. Given the complexity of assessing disease activity, in inflammatory disorders it is commonly assessed through validated composite tools integrating clinical manifestations, laboratory markers, and patient- and physician-reported assessments, which are measured longitudinally to guide treat-to-target decisions.^20^^,^^21^

Applied to CF, we propose that disease activity may be understood as the current intensity of residual CFTR dysfunction and its active downstream consequences that contribute to subsequent organ damage and may change in response to treatment. Inflam­mation is therefore a central domain of CF disease activity, but it is neither its sole determinant nor a sufficient measure of activity. Other domains of residual disease activity include residual mucus dysfunction and chronic airway infection. No validated multidimensional disease activity instrument currently exists for CF.

This concept should also be distinguished from disease severity, which reflects the overall multisystem burden of CF at a given time and is strongly influenced by cumulative, often irreversible structural and functional damage across pulmonary and extrapulmonary manifestations, including established bronchiectasis (Figure 2).

**Figure 2:**
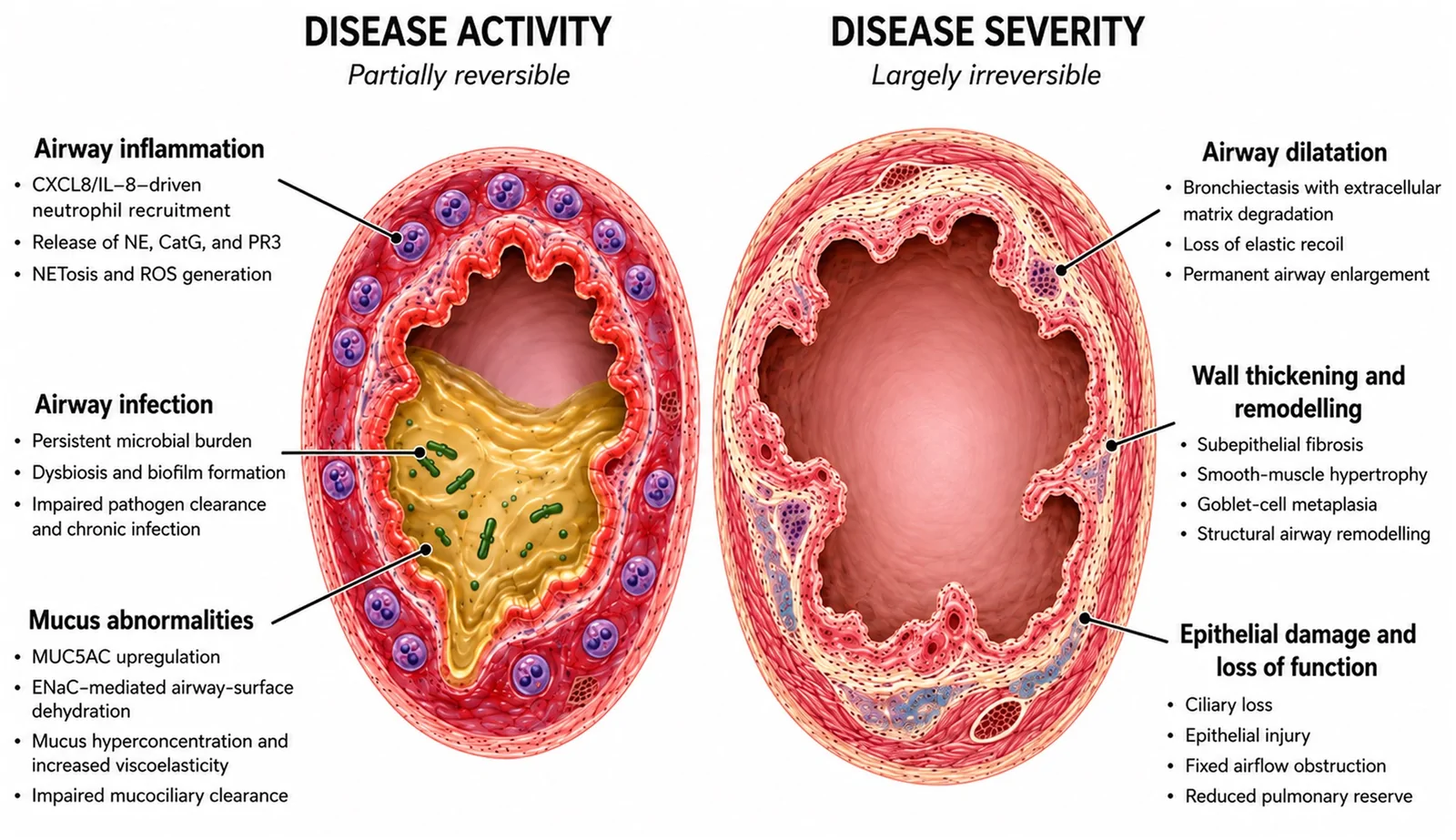
Distinction between disease activity and disease severity in cystic fibrosis lung disease. Disease activity reflects ongoing and partially reversible pathophysiological processes, while disease severity reflects the cumulative, largely irreversible structural and functional consequences of disease. Disease activity may occur at any level of established disease severity. Abbreviations: CatG, cathepsin G; CXCL8, C-X-C motif chemokine ligand 8; ENaC, epithelial sodium channel; IL-8, interleukin 8; NE, neutrophil elastase; NET, neutrophil extracellular trap; PR3, proteinase 3; ROS, reactive oxygen species.

Although conceptually separate, disease activity and disease severity may coexist within the same individual: Active pathophysiological processes may persist in patients with advanced structural damage, while substantial disease severity may remain despite ­limited current activity. Distinguishing disease activity from disease severity is increasingly recognized across respiratory medicine, including chronic obstructive pulmonary disease and bronchiectasis, where it provides a useful framework for understanding risk trajectories and informing therapeutic strategy design.^19^^,^^22^

This distinction is particularly important after initiation of CFTRm. In individuals who start treatment after structural lung disease is already established, posttreatment severity may largely reflect damage accumulated before treatment rather than continued failure to control active disease. These abnormalities may therefore persist despite effective suppression of ongoing disease activity and should not be interpreted as evidence of treatment failure. Conversely, even when CFTRm therapy is initiated early in life, it remains uncertain whether disease processes can be fully prevented. CFTR dysfunction and its downstream consequences may still emerge and could represent a distinct form of RDA occurring in the relative absence of advanced structural damage.

Although a standardized definition of RDA is still lacking in CF lung disease, a range of clinical, radiological, and biological indicators may act as surrogate markers of disease activity across different domains. These may include sputum rheology, radiological detection of mucus plugging and airway wall thickening, and airway inflammation and dysbiosis, as well as patient-reported symptom burden. Such measures have been associated with short-term clinical outcomes, including exacerbation risk and accelerated lung function decline, and may capture key mechanistic drivers of disease progression that are both measurable and potentially amenable to therapeutic intervention.^23^

### Residual disease activity in CF lung disease

As CF is a multisystem disorder, RDA may also occur in extrapulmonary organs, including gastrointestinal, pancreatic, and hepatobiliary. However, the biological manifestations and relevant outcomes are likely to differ across organs and may require domain-specific definitions and validation. The present review therefore focuses primarily on RDA in CF lung disease, while recognizing the need for future work to extend and adapt this framework to extrapulmonary manifestations.

While the impact of RDA on long-term lung disease progression remains uncertain, its persistence suggests that upstream correction of CFTR dysfunction does not fully normalize airway biology. RDA in CF may therefore be conceptualized not only as a marker of ongoing pathology, but also as an obstacle to the restoration of airway homeostasis, which should remain the ultimate long-term goal of treatment.^24^

Importantly, the patterns and magnitude of RDA appear heterogeneous in pwCF, with some patients showing predominant abnormalities in specific domains but minimal involvement in others. In a recent experience of pwCF treated with ETI, changes in sweat chloride concentration (SCC), lung function, body mass index, and patient-reported respiratory symptoms showed only weak correlations with each other, indicating that restoration of CFTR function does not translate uniformly into clinical improvement across all domains of disease.^25^ This framework supports the concept of RDA as a multidimensional phenomenon, where heterogeneous patterns may continue to influence long-term outcomes even in the presence of CFTR restoration. This variability reinforces the need for domain-specific assessment and personalized management approaches.

Because markers of disease activity reflect potentially modifiable processes, their systematic identification and targeted management may help prevent further structural damage and optimize long-term outcomes in pwCF. In the following sections, we review current evidence on RDA with a focus on respiratory domains and propose measurable, clinically meaningful outcomes to inform research priorities and support personalized clinical decision-making.

## Measuring residual disease activity in CF lung disease

### Residual impairment of CFTR activity

CFTR activity is the primary pharmacological target of CFTRm therapy, and SCC is the most widely used surrogate marker. Registrational trials of ETI in adolescents and adults with CF demonstrated substantial reductions in SCC; nevertheless, approximately one-quarter of participants maintained values above the pathological threshold of 60 mmol/L.^5^^,^^6^ Real-world cohorts and patient registry data have confirmed this heterogeneity on a larger scale. In a recent United States–based study, 79% of individuals treated with ETI achieved SCC values <60 mmol/L, whereas 21% remained above this threshold, most frequently among those with Phe508del/minimal function genotypes.^26^ Even the newest generation of CFTRm do not achieve complete normalization of CFTR function. In the recent SKYLINE trials of vanzacaftor/tezacaftor/deutivacaftor (VTD), reductions in SCC appeared greater than those observed with ETI. However, 14% of individuals treated with VTD continued to have SCC values ≥60 mmol/L, compared with 23% in the ETI group. In addition, more than half of participants in both treatment arms exhibited intermediate SCC results (30-60 mmol/L), suggesting persistent incomplete physiological CFTR correction despite treatment.^3^

Residual impairment of CFTR activity may have clinically meaningful downstream implications. First, suboptimal CFTR function does not preclude disease progression over time, as illustrated by the presence of chronic inflammation and bronchiectasis in pwCF with residual function variants.^27^ Zemanick and colleagues further expanded this concept by demonstrating that pwCF with intermediate SCC results after ETI experienced smaller improvements in lung function and exacerbation frequency compared to those achieving SCC <30 mmol/L.^28^

Electrophysiological assessments of CFTR function further reinforced these observations. Although nasal potential difference has been applied in relatively few studies, available data consistently indicate that ETI restores on average approximately 40% to 50% of normal CFTR activity in individuals carrying at least one Phe508del allele, with substantial variability between individuals.^29^

Taken together, these findings indicate that current CFTRm, including ETI and VTD, do not fully normalize CFTR protein function. Instead, residual CFTR dysfunction persists and may act as an upstream driver of ongoing biological activity, warranting further investigation.^29-31^

### Residual impairment of mucociliary clearance

Airway mucus abnormalities remain a key treatable trait in pwCF with established bronchiectasis even after ETI initiation, and their management continues to represent a substantial component of daily treatment burden.^32^ Current standards of care include mucociliary clearance techniques, mucoactive therapies, and inhaled bronchodilators, which aim to facilitate mucus mobilization and provide both therapeutic and preventive benefits in CF lung disease.^33^

Accumulating evidence from the postmodulator era indicates that mucociliary function and sputum viscoelastic properties are not fully restored. Mechanistic studies support this concept. Schaupp and colleagues showed that, despite measurable improvements in sputum rheology after 3 and 12 months of ETI therapy, significant differences persisted compared with healthy controls, indicating incomplete restoration of mucus properties.^32^^,^^34^

Evidence from the PROMISE substudy demonstrates that ETI significantly improved mucociliary clearance (MCC) in 44 patients aged 12 years and older.^35^ On average, whole‑lung MCC over 60 minutes increased from 14.8% at baseline to 22.8% approximately 30 days after ETI initiation. However, the individual responses varied considerably: While many patients experienced marked enhancement in MCC, others showed minimal change, and a subset even exhibited a reduction following treatment. These findings indicate that, although ETI generally enhances mucociliary transport, complete restoration is not consistently achieved across all patients; moreover, in the absence of a healthy control group, the extent to which ETI restores MCC to normal levels remains unclear.

These biological findings are consistent with clinical observations. While ETI initiation is associated with reductions in sputum volume and improved patient-reported mucociliary clearance, a proportion of individuals continue to experience daily sputum production and chronic cough, and mucus remains visible by cross-sectional imaging in many pwCF.^8^^,^^9^ In a survey conducted by the German CF Association, 9% of pwCF reported persistent daily sputum production and 7% ongoing chronic cough despite ETI therapy.^36^

These data suggest that RDA related to airway mucus persists in many individuals and can be detected and quantified through clinical assessment, lung imaging, and advanced functional testing.

Functional assessments provide additional evidence of residual airway disease despite ETI. Normalization in ventilatory inhomogeneity is not universal. In a multicenter pediatric cohort, 14% of pwCF continued to exhibit abnormal lung clearance index values after 12 months of treatment.^18^ Other markers of ventilation inhomogeneity and air trapping, such as Residual Volume / Total Lung Capacity ratio (RV/TLC), also remain abnormal in a subset of individuals and have been associated with attenuated FEV_1_ responses, suggesting that irreversible structural changes may limit functional recovery.^37^

Imaging findings confirm these functional observations. Chronic radiological abnormalities, including established bronchiectasis and lung hyperinflation, showed limited or no improvement in studies using validated imaging scoring systems.^9^^,^^8^^,^^38^ In addition, a recent multicenter French study of adolescents receiving ETI showed progression of bronchial dilatation in 7% of participants, predominantly among those with poorer baseline lung function, indicating that structural deterioration may continue in vulnerable subgroups.^39^

Collectively, these data indicate that, although ETI provides substantial clinical benefit, it does not fully resolve mucus-related airway obstruction or its downstream consequences. Residual abnormalities in mucus properties, ventilation distribution, and air trapping may continue to contribute to ongoing disease activity, particularly in individuals with preexisting structural damage. These observations underscore the importance of continued monitoring and adjunctive therapies aimed at addressing persistent airway dysfunction in the modulator era.

### Residual airway infection

Since the introduction of ETI, growing attention has focused on respiratory microbiology to determine whether and to what extent airway infection patterns change in longitudinal evaluations. Data from the PROMISE-Micro study showed that ETI is associated with a rapid reduction in sputum densities of key CF pathogens within the first month of treatment, followed by long-term stabilization over 3.5 years.^40^ However, complete eradication remains uncommon. Among individuals able to provide adequate sputum samples, clearance of *Pseudomonas aeruginosa* occurred in <10% of cases and, even in patients who became culture negative, the same pathogens were often detectable by PCR months later, suggesting improved host control rather than true microbial eradication.

New infections were also observed. A subset of pwCF who were culture negative for respiratory pathogens prior to ETI developed incident airway infections during follow-up, most frequently with *Staphylococcus aureus* and *P aeruginosa* (approximately 40% and 10%, respectively). Although these events were generally transient and clinically mild, they remain potentially relevant, as they may lead to the establishment of chronic infection or contribute to ongoing airway injury. Persistent infection appears largely driven by preexisting structural lung damage and established chronic infection, with important implications for decisions regarding de-escalation or discontinuation of inhaled antibiotics.

Culture-independent microbiome analyses and longitudinal whole-genome sequencing studies provide additional insight into airway ecology dynamics in the CFTRm era. Across multiple cohorts, ETI consistently reduced total bacterial load and modestly increases microbial diversity.^41^ However, these favorable shifts do not restore a healthy respiratory microbiome. Instead, persistent dysbiosis remains a defining feature after treatment initiation, with airway communities continuing to diverge substantially from those of healthy individuals.^32^ This residual dysbiotic state correlates with markers of ongoing airway inflammation and has been associated with continued lung function decline, indicating that microbial ecology remains an active contributor to RDA despite CFTR modulation.^42^

At the level of individual pathogens, genomic analyses further highlight the durability of chronic infection. Whole-genome sequencing of *P aeruginosa* isolates collected before and after ETI initiation demonstrates remarkable genomic and phenotypic stability, with no consistent evidence of attenuated virulence or reversal of antimicrobial resistance.^43^ These findings suggest that although ETI modifies the airway milieu, it does not exert sufficient impact to disrupt long-established pathogen populations or their adaptive strategies. Rather, ETI appears to induce a shift, but not a complete reset, of the airway microbial ecosystem, which may increasingly resemble that observed in patients with non-CF bronchiectasis.^44^ A better understanding of the evolving interplay between host immunity and microbial networks in the postmodulator era will be essential to inform future microbiome-directed therapeutic strategies.

### Residual airway inflammation

The persistence of pulmonary exacerbations despite CFTRm therapy reflects inflammation-related RDA. Registrational studies reported annualized exacerbation rates ranging from 0.26 to 0.42 per patient-year despite ETI or VTD therapy, events known to have a detrimental impact on prognosis.^3^^,^^5^^,^^45^^,^^46^

Real-world studies evaluating inflammatory biomarkers before and after ETI initiation showed reductions in both systemic and airway inflammation.^32^^,^^47^^,^^48^ However, sputum concentrations of IL-1β, IL-6, and TNF-α, as well as the activity of free and membrane-bound neutrophil elastase (NE), declined but rarely normalized, indicating that airway inflammation persists despite effective CFTR modulation.^32^^,^^34^ Similarly, single-cell transcriptomic analyses of nasal epithelial samples from children aged 6 to 11 years showed that ETI did not completely reverse proinflammatory cellular programs, indicating persistent immune activation despite CFTR correction even in younger individuals.^49^ Transcriptomic analyses of CF airway epithelial cells further support this interpretation. Although neutrophil-associated inflammatory gene expression was markedly downregulated within the first months of ETI therapy in an adult CF cohort, partial reactivation of baseline inflammatory programs emerged over longer follow-up, indicating persistent inflammatory signaling that may influence airway integrity and long-term disease progression.^50^ In a series of studies, NE activity and other neutrophil serine proteases, including proteinase-3 and cathepsin G, tended to decrease to levels comparable to those observed in non-CF bronchiectasis rather than healthy individuals, suggesting a shift toward a bronchiectasis-like inflammatory phenotype characterized by persistent neutrophilic activity.^32^^,^^34^^,^^48^^,^^51^^,^^52^

Although the assessment of local neutrophilic inflammation may represent an important marker of RDA in pwCF, reliable point-of-care methods for its routine evaluation are currently lacking. Biomarkers such as NE may provide a direct measurement of neutrophilic inflammation, but they remain confined to research settings and are not implemented in everyday clinical practice. Previous studies have shown that sputum purulence reflects the presence of neutrophilic airway inflammation.^53^^,^^54^ The green color observed in purulent sputum is largely attributable to myeloperoxidase, an enzyme contained within the azurophilic granules of activated neutrophils. Consequently, assessment of sputum color has been proposed as a simple and accessible surrogate marker of neutrophilic inflammation. In patients with bronchiectasis, sputum purulence correlates with disease severity and clinical outcomes.^55^ Therefore, it may represent a practical risk stratification tool to identify individuals at increased risk of active disease and future exacerbations.

### Residual respiratory symptoms

Patient experience of disease is closely linked to the burden of symptoms. For this reason, registrational trials have consistently included patient-reported outcome measures (PROMs), most commonly the Cystic Fibrosis Questionnaire–Revised (CFQ-R) respiratory domain, among their secondary endpoints.^3^^,^^5^ Considerable heterogeneity in CFQ-R respiratory domain scores has been observed across individuals, with smaller improvements experienced in patients with specific genetic backgrounds.^2^^,^^56^ Despite the increased use of PROMs in clinical trials, data specifically addressing the persistence of residual symptoms remain limited.

A nonnegligible proportion of patients continue to experience symptoms such as cough, mucus production, dyspnea, and chest tightness.^57^ In the phase 3 extension study of ETI, cough remained the most frequently reported event, occurring at a rate of 31 events per 100 patient-years, followed by episodes of increased sputum production (12.7 events per 100 patient-years) and fatigue (11.3 events per 100 patient-years).^58^ In a large survey of 262 pwCF in the United States (78% of whom were receiving CFTRm), the most commonly reported symptoms included lack of energy (74%), cough (73%), and shortness of breath (55%), all of which were associated with variable impacts on quality of life.^59^ These observations suggest that symptom persistence represents an important clinical manifestation of RDA, potentially reflecting ongoing biological processes such as incomplete mucociliary clearance, chronic infection, or persistent inflammation.

Beyond residual symptoms, treatment burden remains a major concern for pwCF in the modulator era and has been identified by patients as a key research priority.^60^ Although several studies have explored the potential for treatment simplification, surveys examining adherence patterns highlight the persistence of substantial treatment burden.^61^^,^^62^

Taken together, these findings indicate that residual symptoms and treatment burden remain important dimensions of RDA in the postmodulator era. Systematic evaluation of these domains, using appropriate assessment tools, may therefore provide valuable insight into the persistence of underlying pathophysiological processes and help guide individualized approaches and treatment optimization in the postmodulator era.

## RDA in personalized clinical management in CF

Introducing the concept of RDA in CF could have important implications for clinical practice, as it offers a framework for tailoring care more closely to the needs of individual pwCF. Identifying domains of ongoing disease activity would allow clinicians to better stratify patients and personalize follow-up strategies. Regular assessment of these domains could help define patient response to therapeutic interventions and guide treatment toward those specific processes that may drive disease progression despite CFTRm.

Different profiles of RDA should not be regarded as fixed categories, but as dynamic phenotypes that may evolve under treatment exposure, adherence, and intercurrent events. A multidomain assessment could therefore help distinguish patients who may benefit from targeted treatment escalation or inflammatory endotyping from those in whom maintenance of current therapy and selective optimization may be more appropriate (Figure 3). Such an approach could also support more standardized decisions regarding treatment escalation or de-escalation. Absence of residual mucociliary clearance impairment may be useful to select the best candidates for treatment simplification, including discontinuation of mucoactive therapies.^63^ On the other hand, a dynamic assessment of RDA domains may help to revise these decisions over time. Importantly, such profiles remain illustrative and hypothesis-generating, and their clinical utility will require prospective validation before they can be used to support treatment decisions.

**Figure 3:**
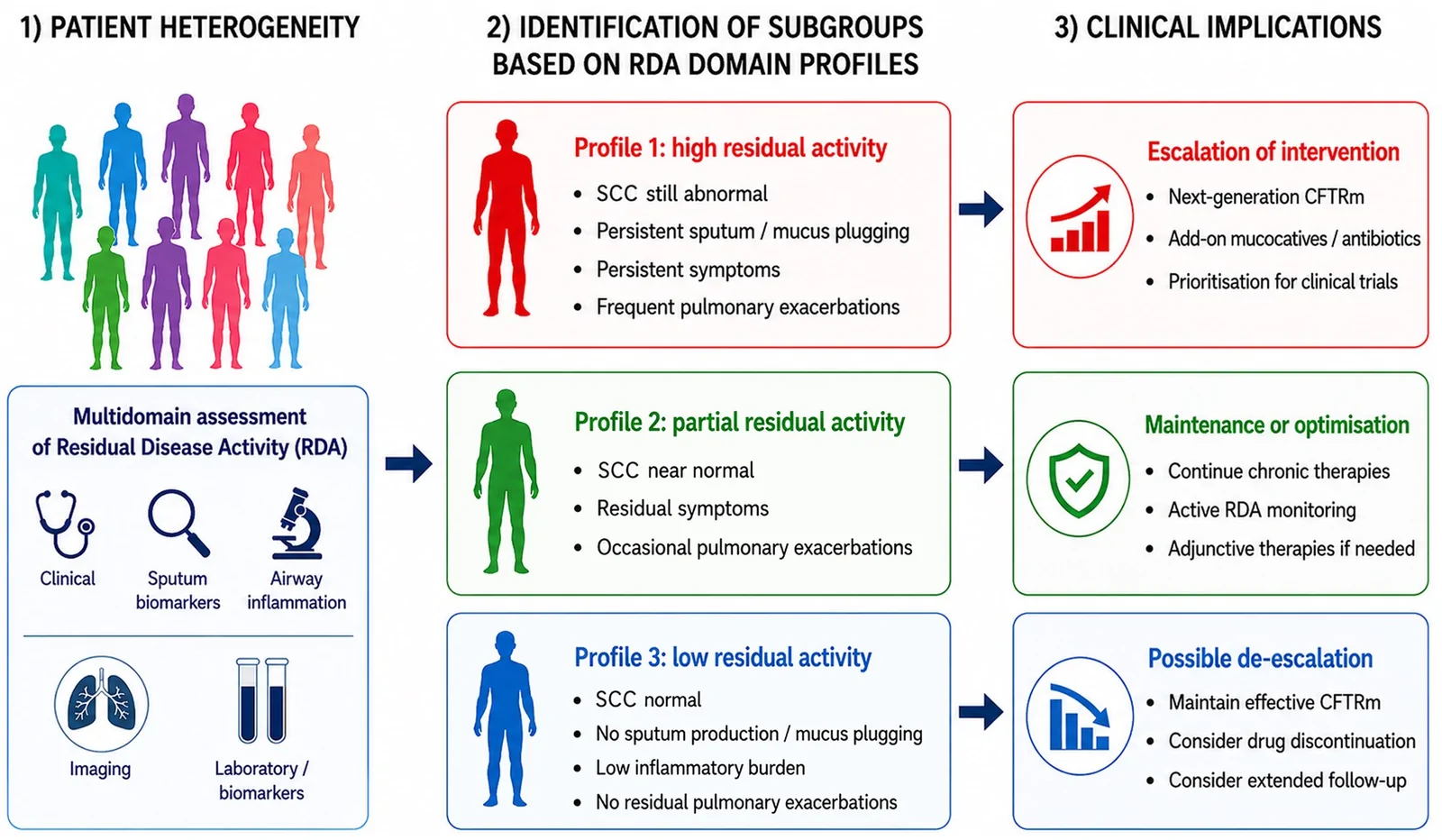
Residual disease activity in cystic fibrosis as a framework for therapeutic stratification and future drug development. Abbreviations: CFTRm, cystic fibrosis transmembrane conductance regulator modulators; RDA, residual disease activity; SCC, sweat chloride.

The availability of reliable markers of RDA could also inform how emerging therapies are evaluated and positioned within the clinical management of CF. As the pipeline of CF-targeted therapies continues to expand, better risk stratification based on measurable domains of RDA may help identify the patients most likely to benefit from novel treatments. Next-generation CFTRm or CFTR amplifier drugs aiming for full restoration of CFTR function could be prioritized for individuals showing persistent CFTR dysfunction despite current therapies.^64^ We may hypothesize that in pwCF carrying predominantly neutrophilic inflammatory signatures, emerging anti-inflammatory therapies such as DPP-1 inhibitors could complement CFTR modulation and help address persistent disease activity.^65-67^ However, more data are needed before any recommendation can be made.

In this way, integrating the concept of disease activity into CF care may help move the field toward a more precise and individualized approach to treatment.

## RDA in clinical research: evidence gaps and future applications

The development of RDA as a clinically informative framework in CF will require a structured, stepwise research program. The initial priority should be to establish a consensus definition of RDA and its core mechanistic domains, while clearly distinguishing active and potentially modifiable processes from largely irreversible damage. This distinction is essential to avoid confusing disease activity with disease severity and to identify the processes most likely to respond to therapeutic intervention. Candidate indicators within each domain should then be evaluated in prospective longitudinal cohorts using repeated and standardized assessments. These studies should determine their feasibility, measurement accuracy, and reproducibility, as well as association with key clinical outcomes, including clinical symptoms, pulmonary exacerbations, disease progression determined from functional decline, and PROMs.

As the direct measurement of several biological processes remains largely confined to research settings, particular emphasis should be placed on developing practical and accessible surrogate measures, including point-of-care biomarkers and imaging-based scores. The most informative and feasible indicators could subsequently be integrated into a provisional multidimensional instrument. Such an instrument would require internal and external validation in independent populations, together with the definition of clinically meaningful change and distinct RDA profiles. Such a staged process would address the principal evidence gaps currently limiting the clinical interpretation of RDA and determine whether it can evolve from a conceptual framework into a reproducible and clinically useful assessment strategy (Table 1). A similar stepwise approach has previously been adopted in systemic lupus erythematosus and rheumatoid arthritis, where multidimensional clinical and laboratory indicators were selected and validated against expert assessment and relevant outcomes to develop composite disease activity indices, such as the Systemic Lupus Erythematosus Disease Activity Index (SLEDAI) and the Disease Activity Score (DAS)/DAS28.^68^^,^^69^

**Table 1:** Domains of residual disease activity and proposed measurement tools.

Domain of RDA	Candidate endpoints	Strengths	Limitations	Trial readiness	Clinical readiness
**CFTR activity**	Sweat chloride concentration	Directly reflects response to CFTR modulation Standardized Highly feasible Familiar to regulators	Poor representation of downstream airway RDA Incomplete correlation with clinical outcomes	High	High
	CFTR functional assays (NPD, ICM)	Mechanistically strong Direct link to CFTR correction Useful in proof-of-concept studies	Limited availability Technically demanding Not scalable for multicenter trials	Low	Moderate
**Mucociliary clearance**	Percent predicted FEV_1_	Well-established Widely available Standardized Highly feasible Accepted by regulators	Insensitive in mild disease Missing early abnormalities Ceiling effect	High	High
	Ventilatory inhomogeneity indices (LCI, RV/TLC)	Increased sensitivity to RDA Biologically plausible for persistent mucostasis Validated endpoint for pediatric studies	Limited availability across centers More technically demanding Longitudinal thresholds still to be defined	Moderate	High
	Sputum production/purulence	Simple and clinically intuitive Relevant to patient experience	Poor standardization Unavailable for many patients on CFTRm Not reflecting biological activity	Moderate	High
	Quantitative chest CT scores	Sensitive to mucus plugging Bridging RDA and future severity	Radiation exposure Higher costs Limited reproducibility	Low	Moderate
	Chest MRI	No ionizing radiation Feasible in children and suitable for repeated measures Can integrate structural and functional information Sensitive to mucus plugging	Longer acquisition times and relatively high cost Not yet fully standardized Lower spatial resolution than CT for lung Restricted to specialized research centers	Moderate	Low
	AI-based imaging analysis	Increased sensitivity and reproducibility Scalable Attractive for RDA	Requires robust validation Limited access Still dependent on imaging acquisition constraints	Low	Low
**Airway infection**	Chronic infection status	Well-established Associated with exacerbation risk	Binary classification Poor sampling after CFTRm	High	High
	Quantitative microbiology	More granularity Measures burden, not just presence/absence	Methodological variability Specimen quality issues Poorly standardized	Moderate	Moderate
	Microbiome-informed monitoring	Detecting organisms missed by routine culture Richer ecological and longitudinal information	Cost Bioinformatic complexity Uncertain interpretation Limited regulatory familiarity	Low	Low
**Airway inflammation**	Sputum neutrophil elastase	Strong mechanistic relevance Directly aligned with neutrophilic inflammation	Poor standardization Sputum collection less feasible after CFTRm	Moderate	Low
	Inflammatory cytokines (eg, IL-6, IL-8)	Biologically informative Useful in defining inflammatory endotypes	Assay variability Uncertain cut-offs Influenced by systemic processes	Moderate	Low
	CRP and selected blood inflammatory markers	Easy to obtain Scalable Inexpensive	Nonspecific Poorly captures localized inflammation	Moderate	Moderate
**Pulmonary exacerbations**	Annual exacerbation rate/time to first exacerbation/severe exacerbations	Well-established Understandable to clinicians Relevant for healthcare utilization Accepted by regulators	Event-driven Less frequent after CFTRm Sensitivity to event rates and follow-up duration Variable thresholds	High	High
**Respiratory symptoms**	CFQ-R respiratory domain	Established PROM Relevant to patient experience	Subjective Possible ceiling effect	High	Moderate
	Cough, sputum, and respiratory symptom scores	Relevant to patient experience	Poor standardization in CF Symptoms may dissociate from underlying biology	Moderate	Moderate
	Treatment burden PROMs	Relevant to patient experience Reflecting RDA in CFTRm era Sensitive to treatment simplification strategies	Indirect measurement of biological disease activity Poor standardization Vulnerable to reporting bias	Moderate	Moderate

Abbreviations: AI, artificial intelligence; CF, cystic fibrosis; CFQ-R, Cystic Fibrosis Questionnaire–Revised; CRP, C-reactive protein; CT, computed tomography; CFTR, cystic fibrosis transmembrane conductance regulator; CFTRm, cystic fibrosis transmembrane conductance regulator modulators; ICM, intestinal current measurement; LCI, lung clearance index; MRI, magnetic resonance imaging; NPD, nasal potential difference; PROM, patient-reported outcome measure; RDA, residual disease activity; RV/TLC, residual volume/total lung capacity.

Once a robust RDA framework and validated assessment tool have been established, the integration into clinical trial design would represent a major research application. Conventional CF trials have frequently relied on broad eligibility criteria based primarily on genotype, age, and lung function, together with endpoints such as percent predicted FEV_1_ and pulmonary exacerbation rates. However, in the CFTRm era, these measures alone may not adequately reflect clinical heterogeneity and low event rates, and RDA-based phenotyping could support a more refined approach to patient stratification and therapy by identifying individuals with measurable abnormalities that can potentially be targeted therapeutically despite otherwise stable conventional clinical measures.^70^ Markers of RDA could therefore be incorporated into eligibility criteria or enrichment strategies, allowing trials to preferentially recruit participants with a clinically relevant unmet need and a greater likelihood of responding to a targeted intervention. RDA domains may inform the selection of more sensitive and mechanism-specific endpoints, reducing reliance on measures that may show limited variability in highly treated populations.

Prospective studies should therefore determine whether incorporating RDA into research may facilitate precision trial design and accelerate the development of therapies targeting disease processes that persist despite CFTR modulation.

## Supplementary Material

aamag370_Supplementary_Data
